# Design and Development of an Antigen Test for SARS-CoV-2 Nucleocapsid Protein to Validate the Viral Quality Assurance Panels

**DOI:** 10.3390/v16050662

**Published:** 2024-04-24

**Authors:** Partha Ray, Melissa Ledgerwood-Lee, Howard Brickner, Alex E. Clark, Aaron Garretson, Rishi Graham, Westley Van Zant, Aaron F. Carlin, Eliah S. Aronoff-Spencer

**Affiliations:** 1 Department of Medicine, Division of Infectious Diseases and Global Public Health, University of California, San Diego, CA 92093, USA; pray@health.ucsd.edu (P.R.); mledgerwood@health.ucsd.edu (M.L.-L.); hbrickner@health.ucsd.edu (H.B.); alexclark@health.ucsd.edu (A.E.C.); agarretson@health.ucsd.edu (A.G.); regraham@health.ucsd.edu (R.G.); westley.vanzant@email.ucr.edu (W.V.Z.); acarlin@health.ucsd.edu (A.F.C.); 2 Department of Pathology, University of California, San Diego, CA 92093, USA

**Keywords:** SARS-CoV-2, nucleocapsid protein, monoclonal and polyclonal antibodies, Enzyme-linked immunoassay, peptide epitope mapping, viral quality assurance, RADx, COVID-19 diagnostics

## Abstract

The continuing mutability of the SARS-CoV-2 virus can result in failures of diagnostic assays. To address this, we describe a generalizable bioinformatics-to-biology pipeline developed for the calibration and quality assurance of inactivated SARS-CoV-2 variant panels provided to Radical Acceleration of Diagnostics programs (RADx)-radical program awardees. A heuristic genetic analysis based on variant-defining mutations demonstrated the lowest genetic variance in the Nucleocapsid protein (Np)-C-terminal domain (CTD) across all SARS-CoV-2 variants. We then employed the Shannon entropy method on (Np) sequences collected from the major variants, verifying the CTD with lower entropy (less prone to mutations) than other Np regions. Polyclonal and monoclonal antibodies were raised against this target CTD antigen and used to develop an Enzyme-linked immunoassay (ELISA) test for SARS-CoV-2. Blinded Viral Quality Assurance (VQA) panels comprised of UV-inactivated SARS-CoV-2 variants (XBB.1.5, BF.7, BA.1, B.1.617.2, and WA1) and distractor respiratory viruses (CoV 229E, CoV OC43, RSV A2, RSV B, IAV H1N1, and IBV) were assembled by the RADx-rad Diagnostics core and tested using the ELISA described here. The assay tested positive for all variants with high sensitivity (limit of detection: 1.72–8.78 ng/mL) and negative for the distractor virus panel. Epitope mapping for the monoclonal antibodies identified a 20 amino acid antigenic peptide on the Np-CTD that an in-silico program also predicted for the highest antigenicity. This work provides a template for a bioinformatics pipeline to select genetic regions with a low propensity for mutation (low Shannon entropy) to develop robust ‘pan-variant’ antigen-based assays for viruses prone to high mutational rates.

## 1. Introduction

The emergence of the coronavirus disease 2019 (COVID-19) pandemic sparked global responses to combat the spread of the severe acute respiratory syndrome virus 2 (SARS-CoV-2). Across these efforts, significant emphasis was placed on developing diagnostic reagents and technologies for detecting the virus. These belong primarily to two classes: Nucleic Acid Amplification Tests (NAAT) to detect the viral genome and Affinity ligands (Antibodies and Aptamers) to detect viral antigenic proteins [1]. NAAT is generally more sensitive than antigen testing; however, the assay requirements for these tests are usually a barrier to their mass deployment. Therefore, antigen-based tests like lateral flow immunoassay (LFIA), which are easy to use and cost-effective, are preferred for point-of-care and home-based testing.

Although some time has passed since the onset of the global pandemic, the development and modification of existing and novel tests have continued to seek improvements in areas including accuracy, cost, and futureproofing against future virus variants [2,3].

In the United States, testing development was supported by the Radical Acceleration of Diagnostics programs (RADx) financed under Operation Warp Speed [4]. These included programs to quickly raise US capacity (RADx-tech), ensure equitable deployment (RADx-UP), and develop the next generation of “radical” diagnostics (RADx-rad) [5]. 

A primary driver for the further development of diagnostic technologies in the RADx-rad program was to develop technologies for tackling the continuing mutability of SARS-CoV-2. Regions within the structural proteins targeted by diagnostic technologies can mutate to diminish recognition or avoid it altogether. This constant mutation has allowed SARS-CoV-2 to remain a public health threat, forcing constant adaptation in tests and treatments that target the evolving regions. This is exemplified by the Spike (S) protein, a common target in therapy; rapid antigen tests; and NAAT due to its outward-facing positioning on the virus’s surface. Although its accessibility and direct role in cellular binding and fusion make Spike a sensible target for recognizing functional virions, frequent mutations in several Spike regions allow it to evade detection and neutralization easily [6,7]. While tests can be modified to account for mutations, tests targeting variable regions of the virus remain relevant only to the specific variants they were developed against.

While variant-specific diagnostics are essential in detecting the virus’s presence and identifying which variant is causing the infection, a more broadly applicable testing method is still needed. It is crucial to trust that a negative COVID-19 test result is due to a lack of the virus rather than the test’s inability to detect an unaccounted-for variant. 

An ideal diagnostic test would target an abundant protein’s region that is sufficiently general to detect the presence of any variant, yet specific enough to avoid detecting other pathogens with similar presentations. Targeting an exposed protein region of the virus that is far less prone to mutations could allow the test to apply to past and future virus variants.

The SARS-CoV-2 nucleocapsid (N) is the most abundant structural protein [8] with historically high conservation, making it a target for many current diagnostics [9]. The N-protein is essential in the virus’s life cycle and is responsible for binding and packaging the viral genome into the ribonucleoprotein complex (RNP) [10]. The protein also plays accessory roles in immune regulation by suppressing viral RNA silencing and assisting in the transcription and replication of viral mRNA [11]. The 419-amino-acid (aa) length N-protein comprises both intrinsically disordered regions (IDRs) and regions with conserved structures, with discrete N-terminal and C-terminal domains present [9,12].

Most FDA-approved, over-the-counter, self-administered, at-home COVID-19 antigen test assays target the N-protein, utilizing its abundance to offer speed and sensitivity [13]. The protein is also highly immunogenic, allowing an overwhelming proportion of antibodies to be raised against it following infection [9,14]. The N-protein serves as a reasonable target due to its decreased tendency for mutation compared to other structural proteins, though it is still susceptible to mutation. It mutates at fewer amino acid positions than the Spike and Envelope (E) proteins, though it does at slightly more positions than the Membrane (M) protein [14,15,16]. Despite its improved reliability, it can still mutate and subvert diagnostic recognition [17]. For instance, mutations in its NTD or linker region can reduce the sensitivity or subvert detection from at-home rapid antigen tests [18]. If mutations in the nucleocapsid protein consistently occur in localized areas, other regions may not see frequent mutations. If areas within the N-protein are conserved between viral variants, they could serve as potential antigenic regions for antibody development in the novel, multi-variant COVID-19 assays.

Observing the mutation rate among viral variants within each open reading frame (ORF) allows for identifying specific amino acid positions or regions that are particularly resistant to mutation. Although many N-protein mutations have been identified within the SARS-CoV-2 variants, most N-protein mutations do not map to the CTD, indicating resistance [19,20]. This indicates the potential of the CTD region as a target for antibody development. These findings can be corroborated using computational methods to identify regions of lower mutability separately.

An unbiased bioinformatics approach to sequence analysis can be implemented to calculate the entropy of regions within the nucleocapsid’s genetic sequence. Regions more prone to mutation will exhibit higher entropy across viral variants, while more conserved regions will exhibit lower calculated entropic values. Low entropy regions can then serve as potential antigenic regions for producing antibodies for diagnostics.

This work describes methods developed by the Diagnostic core of the RADx-rad program to accelerate tests that are sensitive and resistant to mutation while remaining specific enough for clinical utility. Two central roadblocks exist to such development. First, variants for testing may be difficult to obtain, out of circulation, or poorly standardized, and second, the tools for quickly designing reagents targeting constant regions within the viral genome may not be readily available. To navigate this, we developed standardized, inactivated viral quality assurance panels that could be used for test development at lower levels of biohazard containment. At the same time, validating these panels themselves required the rapid development and standardization of quantitative tests to host evolving variants. To achieve this, we employed a hybrid bioinformatic approach that could be automated to detect invariant viral regions preserved in SARS-CoV-2 in a process that can be readily generalized. We herein describe the computational approach for target selection, reagent, and antigen-based assay development, and we demonstrate its efficacy with validated VQAs employed in the RADx program. 

## 2. Materials and Methods

Here, we present a case study of targeting sub-regions of the N-protein. N-protein was chosen due to its abundance and its historical use in cases of antigen-based testing. The process hinges on analyzing the comparative variability of the CTD and NTD regions of the SARS-CoV-2 N-protein. While individual tools allow the assessment of defining mutations [21,22] or amino acid variability [23], there remain no accessible pipelines to quickly conduct flexible variability analysis and comparison at scale. We developed a workflow employing a combination of existing and new tools to facilitate this and similar exploration. This work used genomic sequence data shared via GISAID, the global science initiative [24].

Community Sourced “Defining Mutations”: When navigating the landscape of SARS-CoV-2 mutations, a primary source of insight emerges from examining the consensus SARS-CoV-2 “Defining Mutations”, accessible via the CoVariants website [21]. These mutations signify the phylogenetic root of a variant and can be detected by examining a variant’s root node on a Nextstrain tree [22]. A machine-readable compendium of defining mutations for each variant is also available on the CoVariants GitHub page. We began our investigation into sequence variability by examining these sources.

Shannon Entropy Diversity Metric: Mutation analysis typically incorporates one of three widely accepted metrics for quantifying uncertainty: the Wu-Kabat variability analysis [25], Simpson’s diversity [26], and Shannon entropy [27]. Wu-Kabat variability has been shown to be a biased and less stable metric [28]. Simpson diversity, which is closely related to Shannon entropy, is less sensitive to low-frequency variations [29].

For these reasons, we focus on Shannon entropy. Given a discrete random variable with M distinct realizations and $P_{i}$ the probability of realization i, Shannon’s information entropy (H) is

$$H=-\sum_{i=1}^{M}\;P_{i}\;\log_{2}P_{i},$$

Entropy measures the level of ‘randomness’ or ‘disorder’ within the random process and is widely used across many disciplines [30,31,32,33]. A process with high predictability and low variability has low entropy and vice versa. In biological systems, Shannon’s methods have provided a statistically sound measure of system diversity and sequence analyses [28,34,35,36].

Single Amino Acid Variability: In gene sequence alignments, Shannon entropy has been used to quantify conservative locations by comparing the frequency of amino acid [37] or nucleotide [38] realizations at a given position. For amino acid conservation, related sequences are aligned, and entropy is estimated across the alignment for each amino acid position, j. As the total number of amino acid types is 20, we estimate the Shannon entropy at position j as

H^aj=−∑ i=120kijN log2⁡kijN

where $k_{ij}$ represents the count of amino acid i at position j, and N represents the total number of aligned sequences. Here, the estimated entropy ${\hat{H}}_{aj}$ can range from 0 to 4.322. A value of 0 indicates that only one type of residue is present at that position, signifying complete conservation. On the other hand, a maximum value of 4.322 signifies complete variability, where all 20 types of residues are equally represented at that position [39]. 

Epitope Windowed Shannon Entropy: In our study, we first evaluated the amino acid entropy as the point-based evaluation described above, then considered entropy over an alternate basis to concentrate on short sequences instead of individual residues. That is, we evaluated strings of amino acids that would represent the epitope binding sites. Given that the epitopes typically range in size from four to 12 amino acids long [40], we chose to focus our analysis on an epitope window size of 10 amino acids. The entropy of the window centered at position j is then estimated with:

$${\hat{H}}_{wj}=-\sum_{\;i=1}^{M_{w}}\left(\frac{s_{ij}}{N}\right)\log_{2}\left(\frac{s_{ij}}{N}\right)$$

where $M_{w}=20^{10}$ is the total number of possible amino acid sequences of length 10, and $s_{ij}$ is the count of the jth such sequence at position i among aligned isolates.

This metric, which applies entropy to a window of 10 amino acids, was designed to more accurately gauge the impact of mutations on the linear epitope structure and subsequent antibody recognition. Our approach more faithfully represents the consequences of comprehensive changes within the linear epitope region than single-point mutation analysis.

Sample Collection and Preparation: Sequence analysis in this study used 1800 genomic sequences available on GISAID collected from 20 May 2020 to 5 April 2023. This dataset is accessible at 10.55876/gis8.240302kp and contains 150 sequences each from the 12 WHO variants (e.g., Alpha, Beta, Gamma, Delta, Iota, Mu, Omicron, Eta, Kappa, Lambda, Epsilon, Zeta). The sequences were screened to be ‘complete’ reads, as many genomic data in GISAID are partial sequences. Any sequences containing ‘no read’ sections were eliminated and replaced with alternate samples from GISAID. The complete dataset was further processed by aligning and cropping the sequences to limit the ORF portion of the N-protein. This dataset was then translated into amino acid form. The entire N-protein is 419 amino acids in length, the NTD is 131, and the CTD is 118 amino acids in length, respectively [15]. Only the NTD and CTD amino acids were used for the Shannon entropy analysis. 

Statistics: For our statistical approach, we compared the Shannon entropy values extracted from the N-terminal domain (NTD) and the C-terminal domain (CTD) through a Wilcoxon rank-sum analysis. This non-parametric test is suitable for comparing two independent samples. The Wilcoxon rank-sum analysis enables us to evaluate whether the differences in the entropy values from the two regions are statistically significant, i.e., whether one region exhibits a significantly greater degree of sequence variability than the other. This approach of using a hypothesis test to compare mean entropy values has been used, e.g., to compare mutation frequency across subsequent waves of SARS-CoV-2 in Pakistan [41].

### Cloning and Expression of SARS-CoV-2 Nucleocapsid (Np) Protein Subdomains

The Nucleocapsid protein has two major structural and functional units: the N-terminal domain (NTD) and the C-terminal domain (CTD). The NTD (aa 46–176) is a disordered region responsible for RNA binding, achieving this through a positively charged cavity between the domain’s core and a basic β-hairpin [9,12]. The CTD (aa 247–364) is responsible for dimerization and can also bind with RNA [12]. An IDR linker domain (LKD) (aa 177–246) connects the NTD and CTD, and each domain contains an additional N-terminal arm (aa 1–45) and C-terminal arm (aa 365–419) on its outer edges [11]. We cloned and expressed these domains separately for this study. 

The Np-CTD was expressed from the pUNO1His-plasmid vector (Invivogen, San Diego, CA, USA). Briefly, the target portion of the Np open reading frame (ORF) expressing the residues Np-CTD (Lys248-Pro364) was PCR amplified from the pUNO1His-SARS2-N plasmid vector (4.7 kb) (Invivogen. Catalog code: p1his-cov2-n) using the F1-Forward primer (Xho1 overhang) and the R1- Reverse primer (BamH1 overhang) and ligated to the pUNO1His-plasmid vector, using its Xho1 (Cat. No. R0146S) and BamH1 (Cat. No. R0136S) (New England Biolabs, Ipswich, MA, USA) restriction sites (Supplementary Figure S1). The ligated plasmids were transformed into chemically competent bacterial cells (Subcloning Efficiency™ DH5α Competent Cells Catalog number: 18265017). Individual bacterial colonies were isolated from the Luria Broth (LB) agar plates with a Blasticidin (Invivogen, ant-bl-10p) antibiotic section. Following this, the selected clones were propagated in LB media with Blasticidin, and the plasmid DNA was isolated from the bacterial cells using DNA isolation protocol (Qiagen Miniprep kits, Cat. No./ID: 27104, Qiagen, Hilden, Germany). 

Similarly, the N Terminal portion (Met1-Thr247) of the Np was PCR amplified from the pUNO1His-SARS2-N plasmid vector and subcloned in the pUNO1His-plasmid vector using the F2- Forward primer (Xho1 overhang) and the R2-Reverse primers (BamH1 overhang), using the similar method mentioned above (Table 1).

All the primers were ordered from IDT (Integrated DNA Technologies, Inc., Coralville, Iowa). DNA sequencing was performed on the plasmid DNA vectors from selected clones to ensure the correct ORF sequences. The clones, pUNO1His-SARS2-N, pUNO1His-SARS2-Np-CTD, and pUNO1His-SARS2- Np-NT, were transfected into HEK293 cells following the standard Lipofectamine 3000 Reagent (Invitrogen, Catalog number: L3000001) protocol for the expression of Np-Full-length, Np-CTD, and Np-NT proteins, respectively: (Table 2).

The recombinant proteins were expressed in mammalian cells with a Carboxy terminal Histidine tag (6X-HIS) and secreted into the cell media (conditioned media) (Supplementary Figure S1).

Cell Culture: HEK293 cells obtained from the (ATCC: CRL-1573) were grown in Dulbecco’s Modified Eagle’s Medium, DMEM (Gibco, ThermoFisher Scientific, Waltham, MA, USA. Catalog number: 11965118) media containing 10% Fetal Bovine serum, FBS (Gibco, Catalog number: A5670701) and 1% Penicillin-Streptomycin antibiotics (Gibco, Catalog number: 15140122). VeroE6/TMPRSS2 cells (Sekisui XenoTech) are the VeroE6 cell line modified to express the serine protease TMPRSS2 under Geneticin selection. This cell line is used to produce large stocks of SARS-CoV-2 virus. The cells were grown in DMEM media with 10% heat-inactivated FBS, 2 mM Glutamine, and 1mg/mL of Geneticin (G418). Calu-3 (ATCC: HTB-55) is a lung carcinoma of human epithelial cells used to grow SARS-CoV-2. These cells were grown in DMEM with 1% non-essential amino acids, 2 mM L-glutamine, 1 mM sodium pyruvate, 1.0 g/L glucose, and 20% FBS. All cells were grown at 37 °C under standard tissue culture conditions.

Western Blots: Whole cell lysates, or the conditioned media, were collected from HEK293 cells transiently expressing the Np-FL, Np-CTD, and Np-NT and subjected to the SDS-PAGE technique to resolve the proteins. The proteins were then transferred to a PVDF (Bio-Rad, Hercules, CA, USA. Catalogue: 1620177) membrane using a gel-transfer apparatus and subjected to Western blotting using mouse monoclonal anti-nucleoprotein primary antibodies at 1:1000 dilution. HRP-conjugated goat-anti mouse (ThermoFisher Scientific, Waltham, MA, USA. Catalogue: 31,430) was used as the secondary antibody at 1:1000 dilution, and the antigen-antibody complexes were detected using the ECL system (ThermoFisher Scientific, Waltham, MA, USA. Catalogue: 32,209). HRP-conjugated beta-tubulin (ThermoFisher Scientific, Waltham, MA, USA. Catalogue: MA5-16308-HRP) at 1:4000 dilution was used as the protein loading control.

SARS-CoV-2 isolation and culture: Culture of SARS-CoV-2 variants acquired from BEI (WA1, B.1.351, and B.1.617.2) or isolated from the clinical samples (B.1.1.7, BA.1, BA.2.3, BA.2.12.1, BA.5.1, BF.7, BQ.1, and XBB.1.5). The viruses acquired from BEI were propagated on TMPRSS2-VeroE6 (XenoTech, Kansas City, MO, USA) cells as described previously [42]. The viruses from the clinical samples were isolated as described previously at UC San Diego under IRBs #200477 (B.1.1.7 [42]), #160524 (BA.1 [43], BA.2.3 [43], and BA.5 [44]) and #200236X (BA.2.12.1 [44]). BF.7, BQ.1, and XBB.1.5 were isolated as described for BA.5 under IRB #160524. Briefly, serial dilutions of the clinical sample were serially diluted in DMEM with 1× Pen/Strep + Amphotericin (Anti/Anti) and 10 mM HEPES and applied to monolayers of Calu-3 or TMPRSS2-VeroE6 cells. After one hour, the media above, supplemented with 2% FBS, was added, and the viruses were harvested when the cytopathic effect (CPE) became apparent. Passage 0 stocks were expanded on TMPRSS2-VeroE6 cells and titered by fluorescent focus assay on TMPRSS2-VeroE6 cells. All viral stocks were verified by whole genome sequencing.

All work with the infectious SARS-CoV-2 was conducted in BSL3 conditions at UC San Diego following the guidelines approved by the Institutional Biosafety Committee.

Viral inactivation and VQA panel methods: The viruses, isolated and propagated as described above, were inactivated at BSL3 by UV_254_ irradiation. The UV-inactivation was performed with 400 mJ/cm^2^ in a UVP Crosslinker CL-3000 6.1 (Analytik Jena) in a thin layer of <4 mL in a 10cm dish on a cold block so that the plate bottom was 5.5 inches from the irradiation source. The culture media was similarly UV-treated as a control for downstream assays.

Each SARS-CoV-2 sample was confirmed inactive before its removal to BSL2. The inactivation confirmation was performed by an extended culture of 10% of the volume on TMPRSS2-VeroE6 cells (or TMPRSS2-VeroE6 and Calu3 cells for variant BA.1 due to the reduced infectivity of BA.1 on TMPRSS2-vero) and examination for CPE, followed by the passaging of the entire volume of supernatant to new 96-well plates of cells for additional growth and staining with polyclonal nucleocapsid primary (GeneTex, #gtx135357) and AlexaFluor 594 secondary antibody. The images of whole wells were acquired and examined for positive staining. Positive and negative controls were included at each step.

The inactivated viruses were assembled into viral quality assurance panels (VQAs) by dilution in viral transport medium (VTM) (RMBio VTM-CHT-01L) or media (DMEM + 2% FBS, 1× penicillin/streptomycin, 10 mM HEPES). The dilutions were aliquoted in pre-labeled cryotubes with coded labels and stored at −80 °C until assayed.

Monoclonal and Polyclonal Antibody Production: ProMab Biotechnologies Inc. performed large-scale Np-CTD protein expression and antibody production. The Histidine-tagged Np-CTD protein was expressed in the Mammalian cell expression system (HEK293) and purified using Ni-NTA affinity column chromatography. The purified protein was resolved in SDS-PAGE and stained with Coomassie Blue (Supplementary Figure S1) to check the yield and purity of the protein. Two rabbits and five Balb/c mice were immunized with the protein antigen to generate the rabbit polyclonal and mouse monoclonal antibodies.

The mice were injected with the protein antigen five times for monoclonal antibody production, with an interval of three weeks between each injection to generate the mouse monoclonal antibodies. Next, sera collected from the mice were subjected to direct ELISA using the Np-CTD as the antigen. Based on the ELISA result, the mouse with the highest titer was carried on to hybridoma fusion. The top 10 hybridoma clones (C1–C10) obtained were next tested by direct ELISA using full-length Np. The two best-performing clones (C9 and C10) were selected for monoclonal antibody production and purification. For this, the hybridoma clone cells (C9 and C10) were first expanded in DMEM 10% BSA media using the standard tissue culture technique. The expanded cells were then injected into the peritoneal cavity of five mice through the intraperitoneal (IP)- injection method for antibody generation. The ascites were then collected from the injected mice and purified using an IgG purification column to obtain the monoclonal antibody (mAb 9 and mAb 10).

To generate the rabbit polyclonal antibodies, two rabbits were injected with the Np-CTD protein five times, with an interval of three weeks between injections. After this, the rabbits were sacrificed, their sera were collected, and direct-ELISA was performed using Np-CTD as antigen to test the antibody titer. Then, the sera were precipitated using ammonia persulfate and purified through protein A to get the polyclonal antibody 108.

Enzyme-Linked Immunosorbent Assay (ELISA): The sandwich ELISA was developed using the rabbit polyclonal antibodies to capture the SARS-CoV-2 Np-antigen and the mouse monoclonal antibodies for detection. For this, the 96-well microtiter plates (Sarstedt, North Rhine-Westphalia, Germany, Catalogue: 82.1581.100) were coated with the capture polyclonal antibodies #108 at dilution (1:1000). The antibodies were first diluted in the carbonate-bicarbonate (pH 9.4) buffer (ThermoFisher Scientific Catalogue: 28,382), and 100 µL of this was added to each well. The plates were then sealed with adhesive strips and incubated overnight at 4 °C for the antibodies to bind to the microtiter wells through adsorption. The next day, the contents of the plates were discarded, and the wells were washed twice with 200 µL PBS buffer.

Following this, 200 µL of blocking buffer (1× PBS with 1% BSA) was added to each well, covered with adhesive strips, and incubated at room temperature for two hours. The serum albumin proteins in the blocking buffer used the remaining well-surface that was unoccupied by the antibodies and thus improved the assay’s sensitivity by reducing the background signal and increasing the signal-to-noise ratio. Next, the blocking solution was discarded, and 100 µL of the Np-antigen (recombinant full-length SARS-CoV-2 Np, AcroBiosystems: NUN-C5227) or the UV-inactivated viruses at specified concentrations, diluted in the blocking buffer, were added to the antibody-coated wells. The plates were then covered with the adhesive strip and incubated at 37 °C for 90 min. After this incubation, the solutions in the plates were discarded, and the wells were washed four times with 200 µL PBS buffer. Next, 100 µL of the mouse monoclonal detection antibodies (either mAb 9 or mAb 10) at (1: 4400) dilutions in blocking buffer were added to each well, covered with an adhesive strip, and incubated at room temperature for two hours in a rocker. The solutions were discarded, and the wells were washed four times with 200 µL PBS buffer. The horseradish peroxide (HRP)-conjugated goat-anti-mouse secondary antibodies (ThermoFisher Scientific Catalogue: 31,430) at 1:2100 dilution were added to the wells and incubated in a rocker at room temperature for one hour. Following this incubation, the wells were washed with 200 µL PBS buffer four times, and 100 µL of the 3,3′,5,5′-Tetramethylbenzidine (TMB) substrate solution (ThermoFisher Scientific Catalogue: N301) was added to each well and incubated for 15 min. The chromogenic substrate reaction was terminated by adding 100 µL of stop-solution (ThermoFisher Scientific Catalogue: N600), and the plates were scanned at Abs 450 nm wavelength in a Tecan Multimode microplate reader (TECAN Spark^®^. Mannedorf, Switzerland) within 15 min. The stock concentration of rabbit polyclonal antibodies (#108) was 1.3 mg/mL. The mAb 9 and mAb 10 were at 3 mg/mL and 2.2 mg/mL concentrations, respectively.

The microtiter wells were coated with the Np-antigen at the 250 ng/mL concentration overnight at 4 °C using the above-mentioned method to check the antibody titer and select the hybridoma clones (C1–C10) using the ELISA. The assay was performed by first incubating the wells with 100 µL of the cell supernatant of each hybridoma clone (1:10 dilution in blocking buffer) for two hours at 37 °C for 90 min and washing four times in 200 µL PBS buffer. Goat anti-mouse secondary antibodies (1:2100 dilution) were added to the wells and incubated for one hour at room temperature. Following this, the wells were washed, the signals were developed, and the plates were scanned at Abs 450 nm in a Tecan using the earlier method. All the ELISA were conducted in triplicates (*n* = 3) at every concentration for statistical significance and limit of detection (LoD) calculations.

A commercially available Np-ELISA kit (Ray Biotech, Peachtree Corners, GA, USA. COVID-19/SARS-CoV-2 Nucleocapsid Protein ELISA Kit, Catalog Number: ELV-COVID19N) was used to compare our Np-CTD ELISA kit.

Calculations of Limit of Detection (LOD): The limit of detection is defined as the lowest concentration of an analyte in a sample that can be consistently detected with a stated probability, typically at 95% certainty. Based on the guidelines provided by the FDA’s International Committee on Harmonization [45], the LoD is expressed as

$$LOD=\frac{3.3\sigma }{S}$$

where σ is the standard deviation of the blank, and S is the slope of the curve. Concurrently, the limit of quantification (LoQ), which represents the smallest amount or lowest concentration of a substance that can be determined with established accuracy, precision, and uncertainty, is calculated as

$$LOQ=\frac{10\sigma }{S}$$

Current ELISA work on the N-protein determines concentrations in ng/mL. To translate these concentrations into nanomolar (nM), the molecular weight of the N-protein was calculated based on its size of 419 amino acids (46.09 kDa). Because 1 Da = g/mol, this translates to 46,090 ng/nmol. Therefore, the conversion to nM involves dividing the concentration in ng/mL by 46.09 ng.nmole/mL·L, allowing the analyte’s concentration to be expressed in nM.

Immunofluorescence Assay: Calu-3 cells were infected at an MOI of 0.05 and fixed with 4% formaldehyde in PBS 30 min at RT 24 h later. After PBS washes, the cells were permeabilized and blocked with 30 min incubation in 1% BSA and 0.1% Triton X-100 in PBS. The cells were stained with anti-nucleocapsid primary antibody (GeneTex, Irvine, CA, USA. Cat No. gtx135357) or monoclonal and polyclonal anti-N antibodies followed by AlexaFluor 594 or a 647-conjugated secondary antibody (Thermo Fisher Scientific, Waltham, MA, USA) with nuclear counterstain Sytox Green (Thermo Fisher Scientific, Waltham, MA, USA). Images were acquired on an Incucyte SX5 imager at 20× magnification.

## 3. Results

### 3.1. Bioinformatics Approach for Selecting Antigen

(a) Examination of the pre-existing ‘Defining Mutations’:

The defining mutations for the N-protein SARS-CoV-2 were collected from the CoVariants website [21] on 21 October 2022. The variants that were included within the defining mutations were Alpha B.1.1.7, Beta B.1.351, Gamma P.1, Delta B.1.617.2, Delta 21I, Delta 21J, Omicron BA.1, Omicron BA.2, Omicron BA.4, Omicron BA.5, Omicron BA.2.12.1, Omicron BA.2.75, Omicron BQ.1, Kappa B.1.617.1, Eta B.1.525, Iota B.1.526, Lambda C.37, Mu B.1.621, and Epsilon 20C/s:452R*.

**Figure 1:**
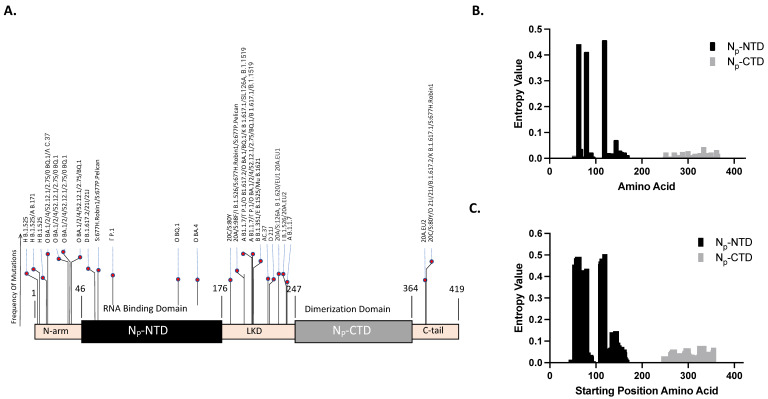
Bioinformatics approach to select the Nucleocapsid protein antigenic region: (**A**) Bar graph illustrating the defining mutations for the N-protein. The location of each mutation is plotted on the *x*-axis, and the height of each bar indicates the number of variants carrying that specific mutation. Notably, no defining mutations are observed in the CTD region. (**B**) Single-point Shannon entropy of the NTD and CTD regions based on 1800 genomic sequences (150 samples per each of the 12 SARS-CoV-2 variants). Each bar represents the entropy value at a specific amino acid position, indicating the variability at that site. (**C**) Windowed Shannon entropy analysis using an epitope-sized window (10 amino acids). Each bar indicates the entropy of the window beginning at the position of the bar on the *x*-axis. In both (**B**) and (**C**), the NTD exhibits higher entropy values compared to the CTD, indicating more variability in the former, as supported by statistically significant *p*-values of <0.005.

These mutation points were plotted with the frequency of mutation representing the number of times this mutation appeared across different variants. In Figure 1A, we illustrate the defining mutations of the N-protein through a bar graph schematic, wherein the mutation locations are indicated, and the heights of the bars correspond to the number of variants that possess that specific mutation. Interestingly, while defining mutations appear in various regions of the N-protein, including the NTD, no defining mutations were observed in the CTD. This implies that the CTD is potentially a highly conserved region, making it a promising target for our antibody development efforts. While these identified mutations provide a helpful starting point, they may only encompass some potential mutations differentiating one variant from another, which requires quantitative mutation analysis such as described here.

(b) Single-Point Shannon Entropy Analysis for Mutation Variability

Next, we compared the ${\hat{H}}_{aj}$, the point-based amino acid entropy estimates over the regions of interest. Figure 1B presents our analysis of our unique dataset of 1800 genomic samples, which includes 150 samples from each of the 12 SARS-CoV-2 variants. Our study focused solely on the NTD and CTD regions, hence the absence of bars in the N-arm, LKD, and C-tail. The bar graph displays the variability at each specific amino acid position (*x*-axis) via entropy values (*y*-axis). The NTD’s three most variable positions were amino acids 119, 63, and 80, with entropy values of 0.458, 0.442, and 0.412, respectively. Conversely, the CTD’s top three positions in terms of variability were amino acids 334, 300, and 288, with entropy values of 0.044, 0.028, and 0.025, respectively. Overall, the NTD exhibits higher average entropy values, indicating more variability, than the CTD—a finding further substantiated by a statistically significant *p*-value of <0.005.

(c) Epitope Windowed Shannon Entropy Analysis

Next, we considered ${\hat{H}}_{wj}$, the estimated entropy over a multiple amino-acid-moving window. In Figure 1C, we display the entropy results derived from our analysis performed over a linear epitope-sized window, in this instance, a 10-amino-acid sequence.

Each bar denotes the entropy or variability across the respective window, with the bar positioned at the first amino acid of the window. This analysis further substantiated our previous findings: the NTD shows higher variability, with the most highly variable three window locations starting at positions 119, 63, and 80, presenting entropy values of 0.504, 0.491, and 0.436, respectively. The CTD, in contrast, displayed the highest values at window starting positions 325, 355, and 296, with entropy values of 0.078, 0.069, and 0.067, respectively. A statistically significant *p*-value of <0.005 confirms the greater variability in the NTD region. This approach more faithfully represents the consequences of comprehensive changes within the entire linear epitope region than single-point mutation analysis.

The code used for data cleaning and entropy calculations can be found on GitHub [46].

### 3.2. Production of Antibodies

Because the Np-CTD was identified by our bioinformatics approach as the region with lesser variability than the NTD, it was selected as the target for antibody development. The open reading frame (ORF) corresponding to the CTD region (Lys248-Pro364) was sub-cloned into the pUNO1His-plasmid vector (Invivogen) for protein expression in mammalian HEK293 cells. We used a mammalian protein expression system to introduce the post-translational modifications (PTMs) on the Np-CTD antigen that would mimic similar modifications during infection [47]. The expressed CTD protein with histidine tag was purified using the standard Ni-NTA affinity purification protocol. The purified protein was resolved in SDS-PAGE and Coomassie stained to determine the purity and yield of the protein (Supplementary Figure S1), and it was used to immunize rabbits and mice to raise polyclonal and mouse monoclonal antibodies.

For polyclonal antibody production, the antibody titer from two immunized rabbits (107 and 108) was compared using the ELISA to select the polyclonal antibody with the stronger CTD binding. The serum samples collected from the immunized animals were tested using purified Np-CTD antigen in the ELISA. The pre-immunized serum samples collected from the same animals were used as negative controls. Compared to the pre-immunized serum, strong antibody titers were detected in the post-immunized serum in both animals, and both animals had comparable polyclonal antibody titers. However, because the serum antibody titer in rabbit 108 was stronger than that in rabbit 107, it was selected for polyclonal antibody purification and subsequent assay development (Supplementary Figure S2).

Five mice were injected with the CTD antigen to produce mouse monoclonal antibodies, and the pre- and post-immunization sera from these animals were analyzed. The mice with the highest serum titer were subsequently selected to produce the hybridoma fusion colonies. Next, the supernatants from 10 hybridoma clones (C1–C10) were analyzed with the ELISA to choose the best monoclonal antibody candidates. Of the 10 hybridoma clone supernatants analyzed, C9 and C10 had the highest titers and were selected for antibody production, purification, and subsequent development of the diagnostics work (Supplementary Figure S3).

### 3.3. Characterization of the Antibodies

(a) Developing sandwich ELISA and determining the assay’s sensitivity

**Figure 2:**
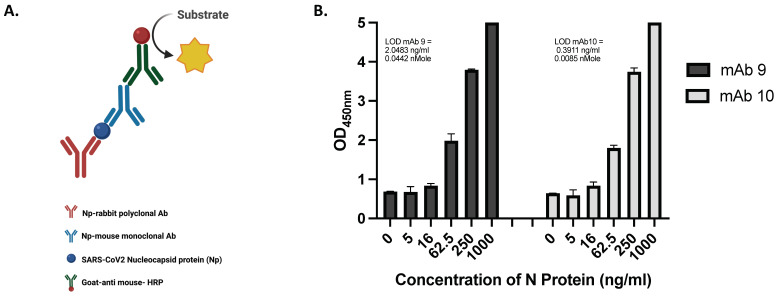

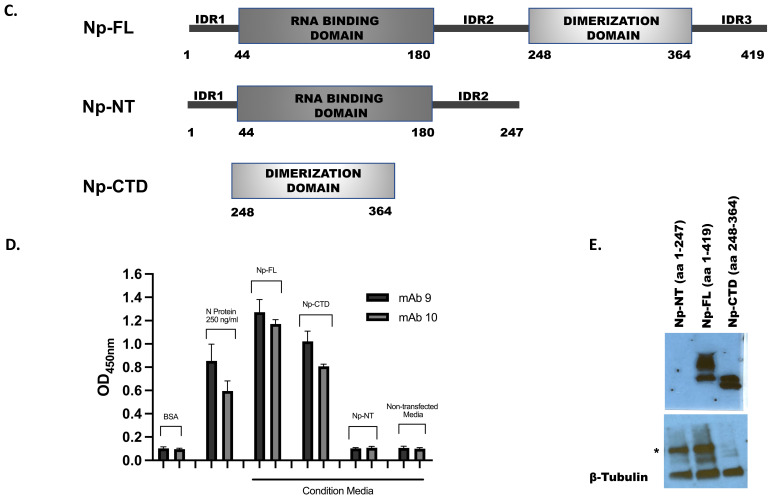
Developing the RADx sandwich ELISA: (**A**) Schema of the ELISA: The rabbit polyclonal antibodies raised against the antigen, Nucleocapsid protein (Np) CTD, were used to coat the microtiter plates for capturing the SARS-CoV-2 Np. The two mouse monoclonal antibodies (either mAb 9 or mAb 10) raised against the antigen Np CTD were used as detection antibodies, and the colorimetric assay was developed using the goat-anti-mouse antibodies conjugated to HRP. The figure was made using BioRender (**B**). Recombinant purified full-length (FL) Np at indicated concentrations were titrated to determine the ELISA’s limit of detection (LoD). All experiments were performed in triplicate sets (n = 3). Data were plotted with error bars denoting the standard deviation of the mean. (**C**) Nucleocapsid protein full length (Np-FL), N-terminal portion (Np-NT), and C-terminal Domain (Np-CTD), as indicated by their size, were cloned and expressed as recombinant proteins in mammalian cells. (**D**) The expressed proteins secreted in the conditioned media were used to determine the specificity of the ELISA. All experiments were performed in triplicate sets (n = 3). Data were plotted with error bars that denoted the standard deviation of the mean. (**E**) The conditioned media were subjected to the Western blots assay using mAb 10. The bands were detected in the Np-CTD and Np-FL; no bands were detected in the Np-NT-conditioned media. The higher migrating bands are due to the post-translational modification of the Np. Beta-tubulin was used as the loading marker; the higher migrating bands, indicated by asterisks, were the Beta-tubulin dimer.

We next developed the ELISA assay using the antibodies produced against the Np-CTD. Figure 2A depicts a schematic representation of the sandwich ELISA developed using rabbit polyclonal and mouse monoclonal antibodies. The rabbit polyclonal antibody, shown in red (108), was immobilized onto the ELISA micro-titer plates to capture purified recombinant Nucleocapsid protein. The mouse monoclonal antibodies, either mAb 9 or mAb 10, depicted in blue, were used for detection. A secondary goat-anti-mouse-HRP antibody was then used for development.

To determine the ELISAs’ sensitivity, titrations of the N-protein were conducted using the mAb 9 and mAb 10-based sandwich ELISAs (Figure 2B). Two independent ELISA experiments were conducted to estimate the limit of detection (LoD), with each test using the same sandwich ELISA technique to account for day-to-day variance. The results of Experiments 1 and 2 for each monoclonal antibody are presented in (Supplementary Table S1). The two experiments for mAb 9 resulted in an average LoD of 1.679 ng/mL (0.036 nMol), while the experiments for mAb 10 resulted in an average LoD of 0.884 ng/mL (0.019 nMol). As the mAb 10-based ELISA exhibited a lower overall LoD, it was identified as the more sensitive assay (Supplementary Table S1).

(b) Determining the Selectivity of the ELISA

In addition to determining the sensitivity of the assays, the specificity was also analyzed to ensure no cross-reactivity or false positives would arise while using the assay. To test for cross-reactivity with other regions of the N-protein, three versions of the nucleocapsid were produced: the full-length N-protein (Np-FL), a truncated version containing the NTD and its flanking IDRs (Np-NT), and a version containing only the CTD (Np-CTD). Schematic representations of the three proteins are depicted in Figure 2C. Plasmids expressing these constructs were transfected into mammalian cells, and the conditioned media containing the secreted proteins were collected. Sandwich ELISAs with the conditioned media were conducted in the same manner as before to compare binding with these three proteins (Figure 2D). Both mAb 9 and mAb 10 exhibited CTD-specificity, binding to the Np-FL and Np-CTD. Binding was not observed in the Np-NT, which showed the same background binding as the BSA control. As a positive control, commercially purchased, purified N-protein (250 ng/mL) was tested, and binding activity was observed to a lesser degree than the Np-FL and Np-CTD. In addition to the BSA and Np-NT negative controls, non-transfected media was also tested, and it exhibited the same degree of background binding as the BSA and Np-NT tests. This indicates that both mAb 9 and mAb 10 are specific to the CTD and are resilient to cross-binding against other regions of the N-protein.

A Western blot was also conducted to corroborate these findings. The conditioned media were probed with mAb 10 antibody. The results of the Western blot are shown in Figure 2E. No bands were observed in the Np-NT sample, while bands were present in the Np-FL and Np-CTD samples. The additional, higher-migrating bands observed in the Np-FL and Np-CTD result from post-translational modifications in the N-protein [48]. β-Tubulin was used as a loading control to verify that each sample was loaded with equal concentrations of the conditioned media. These findings further indicate the CTD-specificity of the assays and the monoclonal antibodies they utilize.

### 3.4. Immunofluorescent Staining of SARS-CoV-2 Infected Cells Using the Antibodies

The ability of antibodies to detect nucleocapsid by immunofluorescence in cells infected with authentic SARS-CoV-2 was also tested. Calu-3 lung cells were infected for 24 h with Omicron sub-variant BA.2.12.1. After fixation, serial dilutions of each antibody were used to stain the infected cells or uninfected controls. Commercial polyclonal anti-nucleocapsid antibody was used as a positive control.

**Figure 3:**
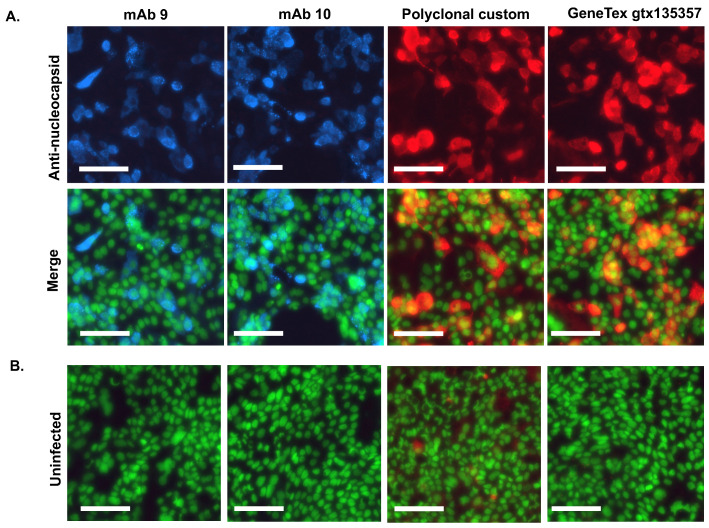
Immunofluorescence assay using the antibodies to detect nucleocapsid in SARS-CoV-2 infected cells: Calu-3 cells infected with SARS-CoV-2 variant BA.2.12.1 were fixed at 24 h post-infection and processed for immunofluorescence. (**A**) Nucleocapsid was detected with monoclonal and polyclonal antibodies at a concentration of 1:100 or commercial polyclonal antibody (GeneTex gtx135357) at 1:1000 (upper panels). Nuclei were counterstained with Sytox Green (lower panels, Merge). (**B**) Uninfected controls were treated as above for each antibody (Uninfected, merge). The scale bar is 100µm.

Both the monoclonal antibodies (mAb 9 and 10) and polyclonal antibodies specifically detected nucleocapsids in infected cells. Some modest background staining was apparent in uninfected cells stained with the polyclonal antibody, but neither monoclonal antibody exhibited background (Figure 3). Monoclonal antibodies gave equal staining intensities. Interestingly, both monoclonal antibodies detected the punctate localization of nucleocapsid proteins; these are the SARS-CoV-2 genome replication foci on the Endoplasmic Reticulum [49].

### 3.5. VQA Testing: Quantitative Evaluation of Variants of SARS-CoV-2

We next evaluated the ELISA’s limit of detection (LoD) using authenticated virus samples. The RADx-DCC program prepared panels of blinded dilutions of UV-inactivated SARS-CoV-2 with known concentrations of N-protein. Each panel included multiple dilutions of UV-inactivated SARS-CoV-2 in at least triplicate.

**Figure 4:**
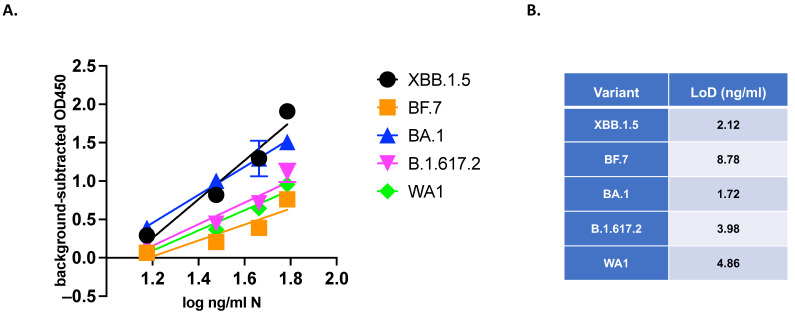
Comparison of ELISA using UV-inactivated SARS-CoV-2 variants: (**A**) SARS-CoV-2 variants XBB.1.5, BF.7, BA.1, B.1.617.2, and WA1 were UV-inactivated, and ng/mL N of stocks was determined by a commercial ELISA. Stocks were normalized to equal ng/mL N and serially diluted in VTM. Triplicate aliquots (n = 3) of each dilution were measured by the ELISA using antibodies by a blinded experimenter. Graphs are mean +/− SD of blank-subtracted OD450 values from triplicate samples. The best-fit line was calculated on log-transformed concentrations in GraphPad Prism 10. (**B**) The LoDs of each variant are listed.

UV-inactivated viruses serially diluted in viral transport medium (VTM) or VTM-only controls were aliquoted and stored at −80 °C in blinded tubes before running the ELISA. A recombinant N standard curve was also run on the plate. The linear range of the dilution curve (log_10_ ng/mL N vs. background-subtracted OD450) was used to produce a best-fit line. The LoD calculated from the recombinant N standard curve was 0.366 ng/mL, and from inactivated XBB.1.5 dilution was 16.7 ng/mL N (Supplementary Figure S4). This result was used to assemble a blinded panel of UV-inactivated SARS-CoV-2 variants of multiple concentrations near the LoD. Measurement by the ELISA demonstrated sensitive detection of all variants tested (Figure 4A). The LoDs of each variant determined by the dilution series are listed in Figure 4B.

**Figure 5:**
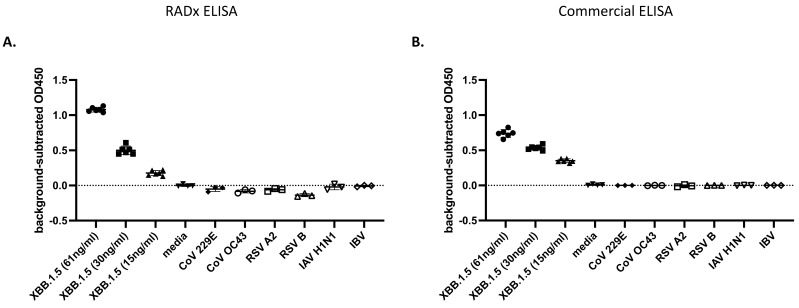
ELISA specificity to SARS-CoV-2: (**A**) SARS-CoV-2 variant XBB.1.5 and additional respiratory viruses were UV-inactivated, diluted in media, and measured by the ELISA. (**B**) An identical dilution series was prepared at the same time and assayed using the Ray Biotech SARS-CoV-2 nucleocapsid ELISA. Both ELISAs were performed blind on non-SARS-CoV-2 (n = 3) and SARS-CoV-2 (n = 6) virus samples. Graphs are mean +/− SD of blank-subtracted OD450 values. The best-fit line was calculated on log-transformed concentrations in GraphPad Prism 10.

Next, potential off-target detection was tested by the ELISA on a blinded panel, including SARS-CoV-2 XBB.1.5, common cold coronaviruses (hCoV 229E, hCoV OC43), respiratory syncytial viruses (RSV A2, RSV B), and influenza viruses (influenza A H1N1, influenza B). Multiple concentrations of XBB.1.5 were detected with a LoD calculated as 2.75 ng/mL N. All non-SARS-CoV-2 viruses were detected at the background level in the ELISA. It should be noted that using a commercial Np-ELISA KIT (Ray Biotech Catalog Number: ELV-COVID19N) comparator for the same challenge panel provided similar results (XBB.1.5 LoD: 2.08 ng/mL N) (Figure 5).

The results were corroborated by a Western blot depicted in (Supplementary Figure S5). Here, bands are visible for all the SARS-CoV-2 variants tested. A lack of visible bands indicates that no binding occurred for the negative distractor viruses. The ELISA and Western blot results strongly suggest the antibodies’ ability to bind to several SARS-CoV-2 variants while lacking cross-reactivity with other distractor respiratory viruses.

### 3.6. Epitope Mapping for the Monoclonal Antibodies

**Figure 6:**
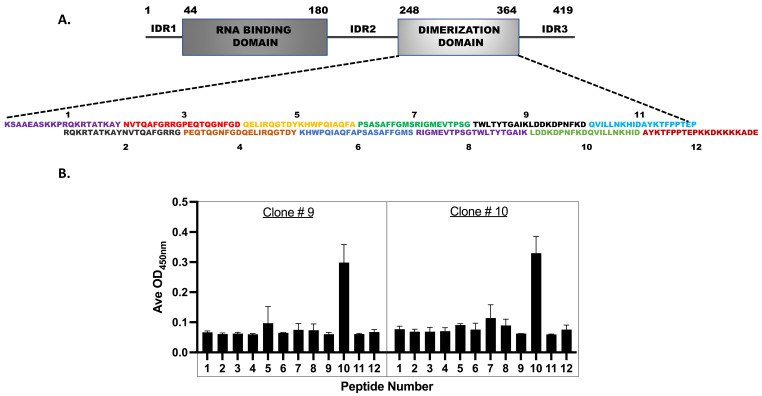

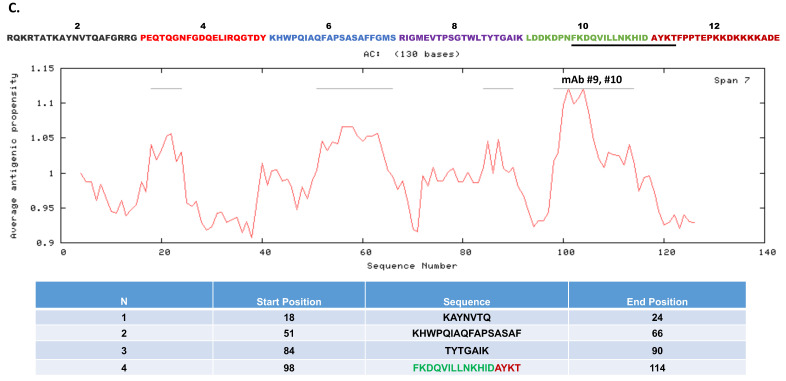
Epitope mapping for the monoclonal antibodies: (**A**) Twelve overlapping peptides spanning the Np-CTD were designed to determine the linear epitopes for the mAb 9 and 10 antibodies. (**B**) The synthesized peptides were subjected to the ELISA using the pepscan method. Peptide #10 demonstrated the highest binding to the mAb 9 and 10, indicating that the major epitope for the monoclonal antibodies is present within these twenty amino acids long linear peptides. All experiments were performed in triplicate sets (n = 3). Data were plotted with error bars that denoted the standard deviation of the mean. (**C**) An in-silico program (http://imed.med.ucm.es/Tools/antigenic.pl) (accessed on 5 December 2023) predicted the Np-CTD’s four major linear epitopes (listed in the table). Notably, the peptide epitope (N = 4) with the predicted highest antigenicity was part of the same peptide #10 determined by our pepscan assay. The predicted epitope spanning peptides 10 and 12 is underscored.

Finally, we wanted to check the Nucleocapsid protein CTD-epitopes to which the monoclonal antibodies (#9 and 10) were binding. We reasoned that because our antibodies could detect the denatured N-protein in Western blots (Figure 2E and Supplementary Figure S5), they were binding to the CTD’s linear epitopes. Therefore, we performed peptide mapping experiments using the pepscan method [50]. For this, we designed 12 overlapping peptides, each of 20 amino-acid lengths, spanning the CTD (Figure 6A), and commercially synthesized them using solid-phase peptide chemistry. The HPLC-purified peptides were then covalently conjugated to the amino group (-NH_2_) of 96-well microtiter plates using the l-ethyl-3-(3-(dimethylamino)-propy1) carbodiimide (EDC) coupling agent provided in the peptide coating kit (Takara, Kusatsu, Japan. Cat No. MK100). The coated peptides were then subjected to the ELISA using the monoclonal antibodies (Figure 6B). The mAb (#9 and #10) bound to only peptide #10; they did not bind to peptides #9 and #11, thus indicating that the epitopes for these antibodies span across the junction of peptides #9 and #11. The data strongly suggest that the two monoclonals (#9 and 10) used in our studies either share the same epitope or that their epitopes are close to each other on this 20 amino acid peptide. In this peptide (339-LDD**K**DP**N**FKD**Q**VIL**L**NKHID-358), the single point Shannon entropy was estimated to be zero at all amino acid positions except at 342, 345, 349, and 353, where modest entropy values of 0.02, 0.007, 0.023, and 0.007, respectively, were estimated. Interestingly, part of this peptide 343-DPNFKD-348 is a major B-cell epitope predicted by the BepiPred algorithm [47]. Additionally, our experimentally determined mAbs binding peptide #10 mapped to one of the Np-CTD’s major antigenic epitopes predicted by an in-silico program (Figure 6C).

Finally, multiple sequence alignment with hierarchical clustering for peptide #10 against the Nucleocapsid protein sequences of other Human coronaviruses (HKU1, OC43, NL63, 229E), Middle East Respiratory Syndrome (MERS), and Severe acute respiratory syndrome coronavirus 1 (SARS 1) identified the mAb 9 and mAb 10′s epitope as a unique sequence (Supplementary Figure S6).

## 4. Discussion

To address the need for continual modifications of SARS-CoV-2 diagnostics due to the mutability of the virus, we aimed to develop an assay that can detect all circulating and historic CoV-2 variants to solve the concomitant problem of providing validated quality assurance panels and supporting test development. Despite several years since the onset of the COVID-19 pandemic, continual development has still been required for diagnostics as the virus mutates to avoid detection and treatment. Rather than having multiple tests to potentially identify only a handful of variants, having one assay whose detection envelops several major variants has epidemiological, logistical, and financial benefits in point-of-care settings. However, developing a test capable of detecting many SARS-CoV-2 variants is a fine line; the assay must be specific enough to detect only SARS-CoV-2 but not so broad as to detect other respiratory viruses that present similar symptoms.

The N-protein was first identified as a starting point for sequence analysis due to its functional importance and immunogenicity. The N-protein is the most abundant structural protein, and antibodies produced against it make up a significant amount of the body’s immune response [9]. The C-terminal domain of the N-protein is particularly immunogenic. Many anti-nucleocapsid antibodies target CTD, and the antibodies produced against this region are especially specific and selective [51]. Considering the importance of the CTD, we separately identified N-protein regions of low mutability utilizing Python-based biostatistical analysis and epitope-windowed Shannon entropy analysis on the N-proteins of major SARS-CoV-2 variants. Because the sequence of the CTD presented significantly lower entropy values than those of the NTD and disordered regions, it was selected as a target for antibody production. We raised several mouse monoclonal and rabbit polyclonal antibodies against this region, and the strongest-binding antibodies were identified using direct ELISA. We used the highest titer candidates to develop sandwich ELISA assays that successfully identified both full-length N-proteins and CTD-containing fragments without cross-binding to fragments lacking the CTD.

We also corroborated our antibodies’ ability to detect the N-protein using immunofluorescent (IF) staining of infected cells. Both our monoclonal and polyclonal antibodies successfully identified the nucleocapsid in infected cells. Our monoclonal antibodies exhibited no background binding to the uninfected cells. A very modest background binding was seen with our polyclonal antibodies; for future IF imaging applications, affinity purification of the polyclonal antibodies using the antigen (Np-CTD) can reduce other non-specific IgGs present and eliminate the background binding.

A primary goal of this study was to optimize and validate blinded viral quality assurance (VQA) panels assembled by the RADx-rad diagnostics core as a generalizable method to produce samples that have clinical equivalency without the need for infected patients and high biosafety containment. While this may be of little concern for slowly mutating pathogens, it can be a significant bottleneck in rapidly spreading and mutating outbreaks such as those experienced in COVID-19. The assay was tested using the panel of inactivated CoV-2 variants and distractor pathogens provided to NIH awardees. The assay detected viral loads in the physiological range (LoD 1.72–8.78 ng/mL). With the same VQA panels provided to awardees, it successfully detected every CoV-2 variant while being resilient to cross-binding with the distractor panel of other respiratory viruses tested (analytic sensitivity and specificity = 100). The variability in the limit of the detection of variants could be explained by differences in epitope availability or structure in our ELISA compared to the commercial ELISA with which the inactivated viruses were quantified.

## 5. Conclusions

In this study, we present a transparent and replicable approach to developing affinity molecules for test validation in a rapidly changing public health emergency. Our combination of heuristics and biostatistical analysis is meant to raise awareness in the scientific community of the need for quality assurance panels as part of the diagnostic development pipeline and the logistical issues of validating a new virus when there are no existing diagnostics. In our study, we identified the C-terminal domain of the SARS-CoV-2 nucleocapsid protein as a potential antigenic region for producing diagnostic antibodies. We utilized both polyclonal and monoclonal antibodies raised against the CTD to develop an ELISA sandwich. We showed that the ELISA could detect several SARS-CoV-2 variants without cross-binding with other coronaviruses and respiratory viruses. We believe future iterations of our assay that substitute our polyclonal capture antibody for a monoclonal antibody will improve the assay’s sensitivity. With the other antibodies produced during the development of this assay, we believe we can create a monoclonal antibody pair with non-overlapping epitopes to optimize capture and detection binding. With these future improvements considered, we believe our assay can serve as a starting point for producing a future multi-SARS-CoV-2 variant test with viability at the point of care testing, such as Lateral Flow Assay.

## Figures and Tables

**Table 1:** Primers used for cloning.

Primers	Sequence	Target
F1	5′-CCGCTCGAGAAGAAATCTGCTGCTGAGGCTTC-3′	Np-CTD (Lys248-Pro364)
R1	5′-CTCGGATCCTTATGGGAATGTTTTGTATGCGTC-3′	Np-CTD (Lys248-Pro364)
F2	5′-AGGCACTCGAGATGTCCGATAATGGGCCACAGAA-3′	Np-NT (Met1-Thr247)
R2	5′-CTCTGGATCCGGTAACGGTCTGCCCCTGTTGCTGTTG-3′	Np-NT (Met1-Thr247)

**Table 2:** Cloned plasmids for protein expression.

Clones	Protein
pUNO1His-SARS2-N (Invivogen Catalog code: p1his-cov2-n)	Full-length Np (Met1-Ala419)
pUNO1His-SARS2-Np-CTD (This work)	Np-CTD (Lys248-Pro364)
pUNO1His-SARS2- Np-NT (This work)	Np-NT (Met1-Thr247)

## Data Availability

The original contributions presented in the study are included in the article and the Supplementary Materials; further inquiries can be directed to the corresponding author.

## Supplementary Materials

The following supporting information can be downloaded at https://www.mdpi.com/article/10.3390/v16050662/s1: Supplementary Figure S1: Cloning, Expression, and purification of Nucleoprotein (Np) C-terminal domain (CTD): The ORF corresponding to the Np-CTD (Lys248- Pro364) was cloned into the vector pUNO1-HIS vector. The cloned vector pUNO1-HIS SARS-CoV-2-Np-CTD was transfected to the mammalian HEK293 cells for protein expression. The Histidine-tagged Np-CTD protein secreted in the cell-conditioned media was purified using Ni-NTA columns. The purified proteins were resolved on the SDS-PAGE to check the purity and yield of the Np-CTD. A major polypeptide band corresponding to the molecular weight of 14 kDa was detected on the Coomassie-stained SDS-PAGE gels; the higher migrating bands are the post-translational modified Np-CTD. Purified Np-CTD was injected into rabbits and mice to produce polyclonal and monoclonal antibodies. Supplementary Figure S2: Production of Np-CTD rabbit polyclonal antibody. The purified Np-CTD antigen was injected into two rabbits (Rb 107 and 108) to produce the polyclonal antibodies. The serum collected pre- and post-immunization (Bleed 3) from the animals was tested by ELISA using the purified Np-CTD proteins. Both Rb-107 and Rb-108 demonstrated very high antibody titers compared to the pre-immunized serum. Rb-108 polyclonal Ab was selected to develop the subsequent sandwich ELISA. All experiments were performed in triplicate sets (n = 3); data were plotted with error bars that denoted the standard deviation of the mean. Supplementary Figure S3: Production of Np-CTD mouse monoclonal antibody: The hybridoma clones’ supernatant (C1-C10) obtained from the mouse injected with the purified Np-CTD antigen was tested by ELISA using the purified Np proteins. Clones 9 and 10 demonstrated the highest reactivity in ELISA, and the purified monoclonal antibodies mAb 9 and mAb 10 from these clones were selected to develop the subsequent sandwich ELISA. The purified Np was titrated at the indicated concentrations (ng/mL) in parallel for the positive control. All experiments were performed in triplicate sets (n = 3). Data were plotted with error bars that denoted the standard deviation of the mean. Supplementary Figure S4: Estimating ELISA’s Limit of Detection (LoD) for UV-inactivated SARS-CoV-2 XBB.1.5: A dilution series of UV-inactivated SARS-CoV-2 XBB.1.5 was prepared in viral transport media and stored at −80 °C. (A) Samples were prepared in triplicate and measured by ELISA using antibodies by a blinded experimenter. (B) Recombinant N-protein was serially diluted and included in the ELISA in A. Graphs are mean +/− SD of triplicate samples. The OD values of blanks were subtracted, and the best-fit line was calculated on log-transformed concentrations in the GraphPad Prism 10. Supplementary Figure S5: Western blot to check the specificity of the antibody: The UV-inactivated SARS-CoV-2 variants (WA1, B.1.1.7, B.1.351, B.1.617.2, BA.1, BA.2.12.1) and other distractor respiratory viruses (HCoV-OC43, HCoV-299E, RSV A2, RSV B) were resolved in SDS-PAGE and subjected to the Western blot assay using mAb 10. Single polypeptide bands corresponding to the molecular weight of Np (~45 kDa) were only detected with the SARS-CoV-2 variants. No bands were detected for the distractor viruses. Beta-tubulin was used as the loading control; the higher migrating bands indicated by asterisk are the tubulin dimer. Supplementary Figure S6: Phenogram display from the sequence alignment of monoclonal antibody epitope against Nucleocapsid proteins of other coronaviruses: Multiple sequence alignment with hierarchical clustering was performed for the mAb 9 and mAb 10 epitope (peptide#10) against the Nucleocapsid protein sequence of human coronaviruses (HKU1, OC43, NL63, 229E), Middle East Respiratory Syndrome (MERS), and severe acute respiratory syndrome coronavirus 1 (SARS 1) using Multalin version 5.4.1. The minimum distance between sequences in this Phenogram in Point Accepted Mutation (PAM) is set at 20. Supplementary Table S1: Limit of Detection (LoD) and Limit of Quantitation (LoQ) for RADx-ELISA.
