# Supplementary material for: Design and Development of an Antigen Test for SARS-CoV-2 Nucleocapsid Protein to Validate the Viral Quality Assurance Panels
Source: Viruses. 2024 Apr 24;16(5):662. doi: 10.3390/v16050662 (PMC11125937; doi:10.3390/v16050662)
Supplement: Supplementary file 1 [file viruses-16-00662-s001.zip › viruses-2965548-supplementary.pdf]

Supplementary Materials

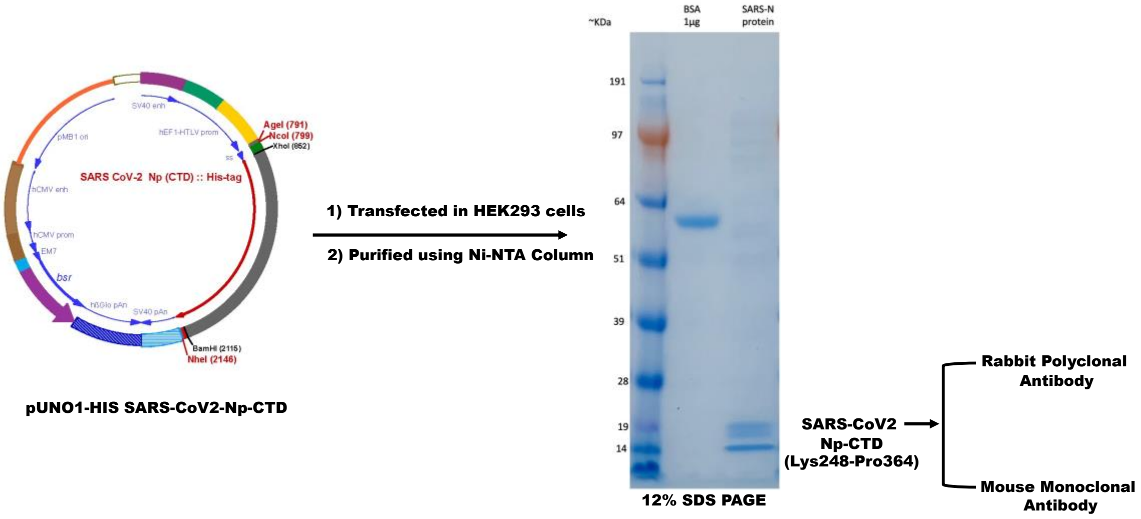

Figure S1. Cloning, Expression, and purification of Nucleoprotein (Np) C-terminal domain (CTD): The ORF corresponding to the Np-CTD (Lys248- Pro364) was cloned into the vector pUNO1-HIS vector. The cloned vector pUNO1-HIS SARS-CoV-2-Np-CTD was transfected to the mammalian HEK293 cells for protein expression. The Histidine-tagged Np-CTD protein secreted in the cell-conditioned media was purified using Ni-NTA columns. The purified proteins were resolved on the SDS-PAGE to check the purity and yield of the Np-CTD. A major polypeptide band corresponding to the molecular weight of 14 kDa was detected on the Coomassie-stained SDS-PAGE gels; the higher migrating bands are the post-translational modified Np-CTD. Purified Np-CTD was injected in rabbits and mice to produce polyclonal and monoclonal antibodies.

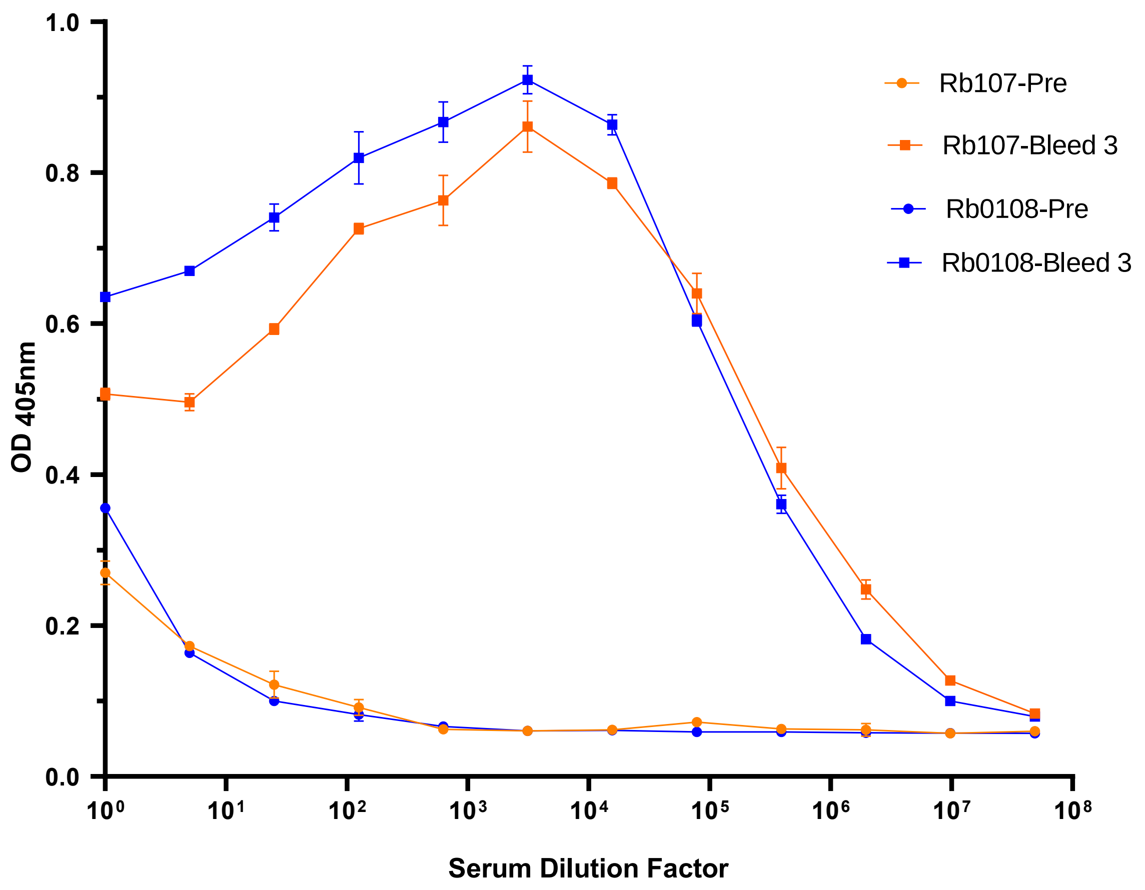

Figure S2. Production of Np-CTD rabbit polyclonal antibody: The purified Np-CTD antigen was injected into two rabbits (Rb 107 and 108) to produce the polyclonal antibodies. The serum collected pre- and post-immunization (Bleed 3) from the animals was tested by ELISA using the purified Np-CTD proteins. Both Rb-107 and Rb-108 demonstrated very high antibody titer compared to the pre-immunized serum. Rb-108 polyclonal Ab was selected to develop the subsequent sandwich ELISA.

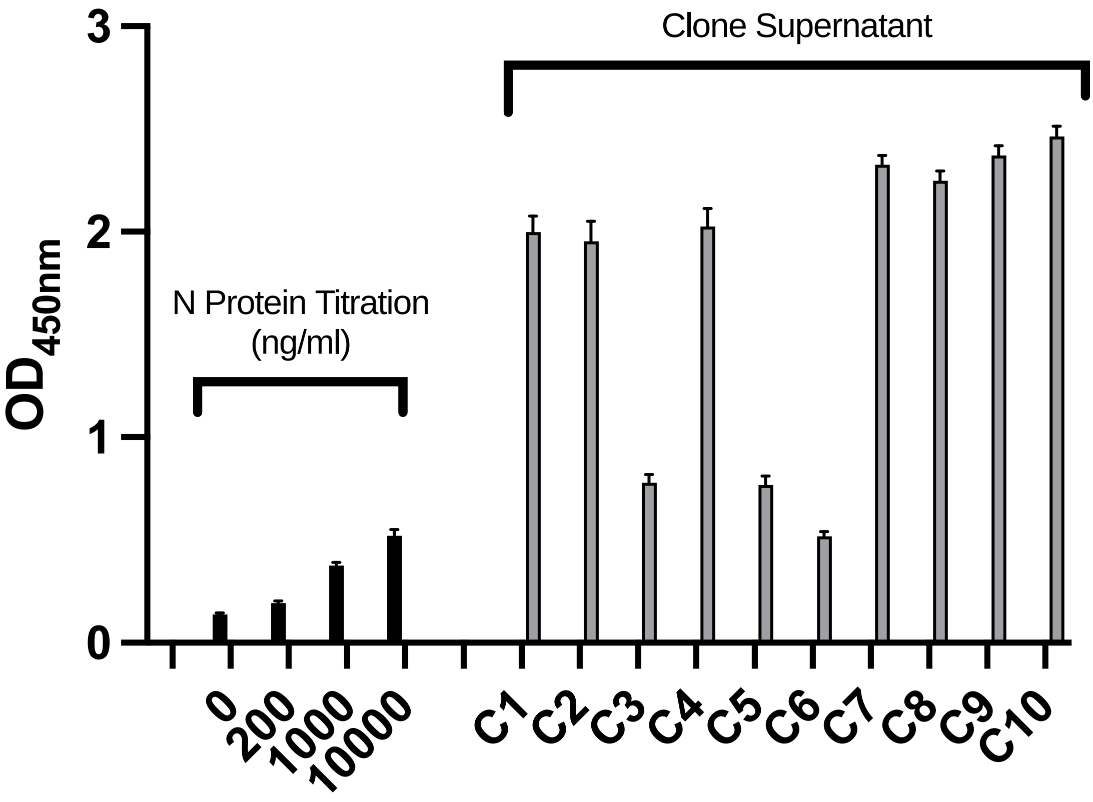

Figure S3. Production of Np-CTD mouse monoclonal antibody: The hybridoma clones' supernatant (C1-C10) obtained from the mouse injected with the purified Np-CTD antigen was tested by ELISA using the purified Np proteins. Clones 9 and 10 demonstrated the highest reactivity in ELISA, and the purified monoclonal antibodies mAb 9 and mAb 10 from these clones were selected to develop the subsequent sandwich ELISA. The purified Np was titrated at the indicated concentrations (ng/ml) in parallel for positive control.

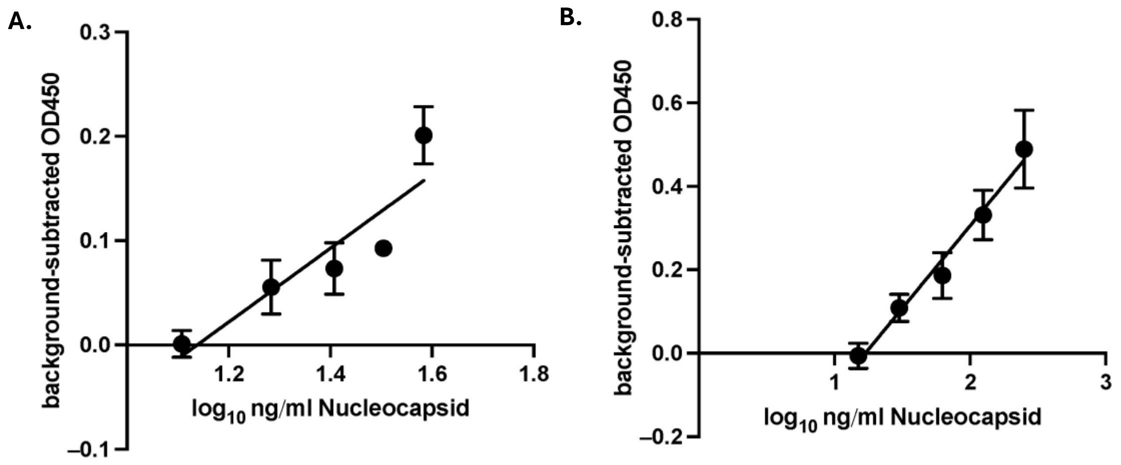

Figure S4. Estimating ELISA's Limit of Detection (LoD) for UV-inactivated SARS-CoV-2 XBB.1.5: A dilution series of UV-inactivated SARS-CoV-2 XBB.1.5 was prepared in viral transport media and stored at -80°C. **A.** Samples were prepared in triplicate and measured by ELISA using antibodies by a blinded experimenter. **B.** Recombinant N protein was serially diluted and included in the ELISA in **A.** Graphs are mean +/- SD of triplicate samples. OD values of blanks were subtracted, and the best-fit line was calculated on log-transformed concentrations in GraphPad Prism 10.

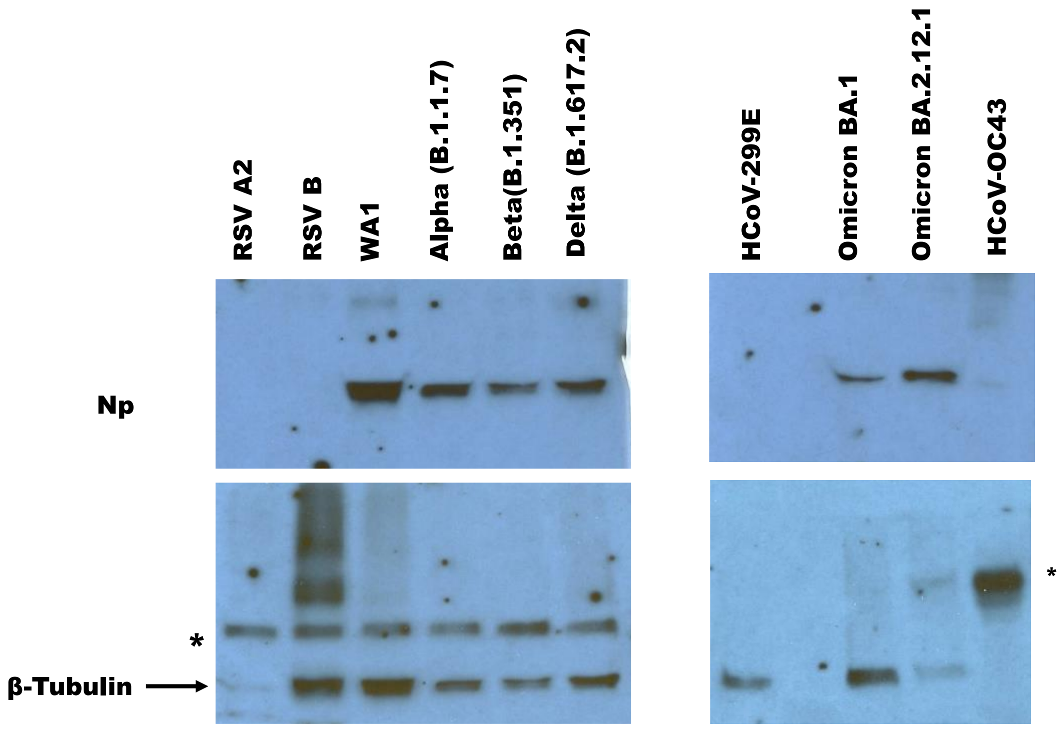

Figure S5. Western blot to check the specificity of the antibody: The UV-inactivated SARS-CoV-2 variants (WA1, B.1.1.7, B.1.351, B.1.617.2, BA.1, BA.2.12.1) and other distractor respiratory viruses (HCoV-OC43, HCoV-299E, RSV A2, RSV B) were resolved in SDS-PAGE and subjected to the Western blot assay using mAb10. Single polypeptide bands corresponding to the molecular weight of Np (~45 kDa) were only detected with the SARS-CoV-2 variants. No bands were detected for the distractor viruses. Beta-tubulin was used as the loading control; the higher migrating bands indicated by Asterix are the tubulin dimer.

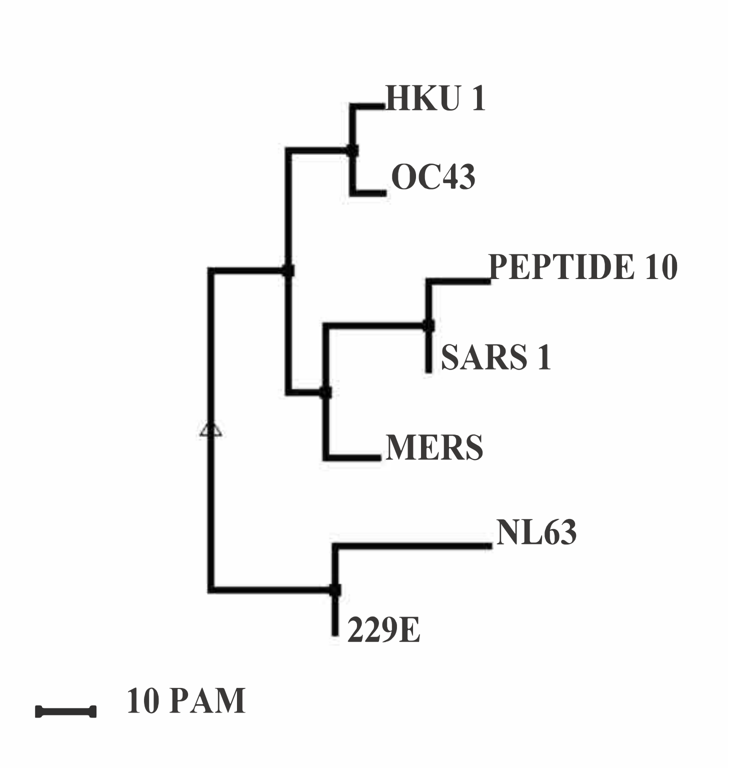

Figure S6: Phenogram display from the sequence alignment of monoclonal antibody epitope against Nucleocapsid proteins of other Coronaviruses: Multiple sequence alignment with hierarchical clustering was performed for the mAb9 and mAb10 epitope (peptide#10) against the Nucleocapsid protein sequence of Human coronaviruses (HKU1, OC43, NL63, 229E), Middle East Respiratory Syndrome (MERS), and Severe acute respiratory syndrome coronavirus 1 (SARS 1) using Multalin version 5.4.1. The minimum distance between sequences in this Phenogram in Point Accepted Mutation (PAM) is set at 20.

mAb 9	Experiment 1	Experiment 2	Average	SD
Slope	0.125	0.050	0.088	0.053
SD of Blank	0.008	0.002	0.005	0.004
LOD (ng/ml)	2.048	1.310	1.679	0.522
LOD (nMol)	0.044	0.028	0.036	0.011
LOQ (ng/ml)	0.621	0.397	0.509	0.158
LOQ (nMol)	0.013	0.009	0.011	0.003

mAb 10	Experiment 1	Experiment 2	Average	SD
Slope	0.125	0.154	0.140	0.020
SD of Blank	0.001	0.007	0.004	0.004
LOD (ng/ml)	0.391	1.377	0.884	0.697
LOD (nMol)	0.008	0.030	0.019	0.015
LOQ (ng/ml)	1.185	4.589	2.887	2.407
LOQ (nMol)	0.026	0.100	0.063	0.052

Table S1: Limit of Detection (LoD) and Limit of Quantitation (LoQ) for RADx-ELISA.
